# Fatty Acid-Binding Proteins and Substance Use Disorders: From Lipid Signaling to Therapeutic Targets

**DOI:** 10.3390/genes17091000

**Published:** 2026-08-25

**Authors:** Aidan Powell, Noa Yamaguchi, Mariana Delgado, Kenneth Blum, Albert Pinhasov, Igor Elman, Panayotis K. Thanos

**Affiliations:** 1 Behavioral Neuropharmacology and Neuroimaging Laboratory on Addictions (BNNLA), Clinical Research Institute on Addictions, Department of Pharmacology and Toxicology, Jacobs School of Medicine and Biomedical Sciences, University at Buffalo, Buffalo, NY 14203, USAnoayamag@buffalo.edu (N.Y.); md248@buffalo.edu (M.D.); 2 Division of Addiction Research and Education, Center for Sports, Exercise and Global Mental Health, Western University Health Sciences, Pomona, CA 91766, USA; 3 Department of Molecular Biology, Adelson School of Medicine, Ariel University, Ariel 407000, Israel; albertpi@ariel.ac.il (A.P.);; 4 Cambridge Health Alliance, Harvard Medical School, Cambridge, MA 02115, USA

**Keywords:** fatty acid-binding proteins, FABPs, endocannabinoid system, substance use disorders, reward deficiency syndrome, addiction, dopamine

## Abstract

Fatty acid-binding proteins (FABPs) are a family of intracellular lipid chaperones that transport fatty acids and other hydrophobic molecules, playing essential roles in cellular lipid metabolism, signaling, and brain function. Within the central nervous system, FABP3, FABP5, and FABP7 facilitate the trafficking of long-chain polyunsaturated fatty acids and endocannabinoids, thereby modulating key regulatory pathways including the endocannabinoid system (ECS), peroxisome proliferator-activated receptor (PPAR) signaling, and dopaminergic neurotransmission. Peripherally, FABP1 and FABP4 contribute to hepatic drug metabolism, kidney excretion, and inflammatory processes in both tissues, with implications for the pharmacokinetics of substances of abuse. This narrative review synthesizes the current literature on FABPs and their involvement in substance use and addiction-related behaviors. Evidence from transgenic knockout models, pharmacological inhibition studies, and adeno-associated virus vector approaches demonstrates that manipulation of FABP subtypes can alter reward-related behaviors across multiple substances, including THC, ethanol, nicotine, and cocaine. Reduction or knockout of FABP7 alters THC metabolite levels in a sex-dependent manner. FABP3 shows involvement with dopamine receptor expression; however, interaction between FABP3 modulation and specific substances has sparsely been investigated. FABP5 has vastly diverging interactions with addictive behavior and appears to be substance dependent, as downregulation reduces cocaine self-administration, but knockout enhances nicotine conditioned place preference (CPP) and increases brain uptake of THC. Combined deletion of FABP5 and 7 additionally reduces cocaine CPP and reinstatement, while showing promising decreases in ethanol consumption paradigms. FABPs may be a potential therapeutic target for treating substance use disorders and underlying reward deficiency mechanisms underlying addiction and further research is required to elucidate specific mechanistic effects and eliminate potential adverse consequences of chronic FABP modulation.

## 1. Introduction

### 1.1. What Are Fatty Acid-Binding Proteins?

Fatty acid-binding proteins (FABPs) are a group of lipid chaperones involved in the membrane and intracellular transport of fatty acids and other hydrophobic molecules, making them essential regulators of cellular lipid metabolism [1]. By transporting long-chain polyunsaturated fatty acids (PUFASs), FABPs support brain development and neurotransmission. Alterations in PUFA levels or FABP expression can modulate key regulatory pathways such as peroxisome proliferator-activating receptors (PPARs), retinoid X receptors (RXRs), and the endocannabinoid system (ECS) [2]. These changes can cause aberrant neurodevelopment and various neuropsychiatric disorders [3]. In addition, FABPs may exert systemic effects beyond their sites of expression, with circulating levels often reflecting the physiological state of their tissues of origin [4].

The FABP family consists of 12 proteins, with ten being found in humans: FABP1, FABP2, FABP3, FABP4, FABP5, FABP6, FABP7, FABP8, FABP9, and FABP12 [5]. Although FABPs share a similar structure and ligand-binding capacity, their variation in protein–protein and protein–membrane interactions influence their distinct roles within cells [6]. A summary of the role of FABP subtypes in biophysiology can be seen in Table 1.

FABP subtypes exhibit distinct localization patterns across tissues and cell types. FABP1 (liver FABP) is highly expressed in the liver, and FABP2 (intestinal FABP) is localized to the small intestine, where it is co-expressed with FABP1 [4,7]. FABP3 (heart FABP) is highly expressed in cardiac and skeletal muscles as well as in the brain, particularly in dopaminergic neurons [8,9]. FABP4 (adipocyte FABP) is primarily expressed in adipocytes, macrophages, monocytes, and endothelial cells, making it a marker of adipocyte maturation [4]. Similarly, FABP5 (epidermal FABP) is present in neurons, astrocytes, adipocytes, macrophages, dendritic cells, and airway epithelia, but is also abundantly expressed in the epidermis [10]. FABP6 (ileal bile acid-binding protein) is mainly present in the ileum, which is the site of bile acid uptake [4]. FABP7 (brain FABP) is expressed in astrocytes and radial glial cells, whereas FABP8 (myelin FABP) is a protein of myelin in the central nervous system (CNS) and Schwann cells in the peripheral nervous system [11,12]. FABP9 is found in the salivary gland and mammary gland as well as the testis, while FABP12 is expressed in both the testis and retina [4,13].

FABPs participate in a range of physiological processes related to lipid transport and cellular regulation, with functions varying between isoforms. FABP1 is involved in hepatic and intestinal lipid metabolism and has been shown to influence whole-body homeostasis, in part through regulating endocannabinoid levels. This is demonstrated in FABP1 knockout models showing decreased body weight, reduced hepatic steatosis, and altered fatty acid ethanolamide metabolism [14,15]. In contrast, FABP2 is associated with intestinal integrity and permeability, with circulating levels used as a marker of intestinal damage [7,16]. FABP3 supports fatty acid oxidation in highly metabolic tissues, while also playing a role in neuronal signaling, particularly through dopaminergic pathways [9,17]. More broadly, FABP4 and FABP5 link lipid metabolism with inflammatory and systemic signaling processes, influencing immune responses, metabolic disease, and cancer-related pathways [18,19,20]. FABP6 is involved in bile acid trafficking and participates in regulatory pathways that coordinate lipid and bile acid homeostasis [21]. Within the CNS, FABP3, FABP5, and FABP7 further integrate lipid transport with signaling by facilitating the handling of long-chain PUFAs, which are essential for brain development, synaptic plasticity and neurotransmission [1,9,22]. Through this interaction, they engage in key pathways including PPAR signaling, RXRs activation, and the ECS modulation, thereby linking lipid availability to gene expression and neuronal activity [1]. In particular, FABP7 is strongly associated with neurodevelopmental processes such as neuronal migration and astrocyte function [23]. In contrast, FABP8 contributes to myelin structure and neural integrity, and FABP9 is associated with reproductive and disease-related processes [12,24,25]. FABP12, a more recently identified isoform, has been linked to cancer progression through PPARγ signaling and altered fatty acid utilization [13]. These processes and downstream effects of FABPs and the modulation of such may produce favorable effects regarding reward behaviors. Potentially, with further research, these mechanisms can be further elucidated and effects specific to addiction can be harnessed. In this review, promising mechanistic links and tissues specific effects, as well as promising current data specific to drug-seeking and consumption behaviors on various drugs of abuse will be discussed.

**Table 1:** FABP protein-specific roles in biophysiology. FABP, fatty acid-binding protein; CNS, central nervous system; PNS, peripheral nervous system; PUFAs, polyunsaturated fatty acids; PPARs, peroxisome proliferator-activated receptors; RXRs, retinoid X receptors; ECS, endocannabinoid system. Regions expressed in the brain noted at the bottom of the table.

FABP Protein	Location	Roles/Mechanisms and Functional Relevance	References
**FABP1**	Liver, intestine	Regulates lipid metabolism and endocannabinoid levels Influences whole-body homeostasis	[14,15]
**FABP2**	Small intestine	Helps regulate intestinal integrity and permeability Serum levels dictate intestinal damage	[7,16]
**FABP3**	Heart, skeletal muscle, brain	Supports fatty acid oxidation and dopaminergic signaling Links lipid metabolism with neural signaling	[9,17]
**FABP4**	Adipocytes, macrophages	Lipid metabolism, inflammatory signaling Potential serum marker of tissue-specific metabolic disease and immune-related processes	[4]
**FABP5**	Skin, brain, immune cells	Lipid metabolism, inflammatory signaling Plays role in immune function, cellular senescence, and nuclear factor-kappa B signaling in cancer	[18,19,20]
**FABP6**	Ileum	Bile acid trafficking Coordination of lipid and bile acid function	[21]
**FABP7**	Brain (astrocytes, radial glia)	Neuronal migration and astrocyte function Neurodevelopmental processes	[23]
**FABP8**	Myelin (CNS & PNS)	Contributes to myelin structure Supports neural integrity	[24,25]
**FABP9**	Salivary gland, mammary gland, testis	Expressed in reproductive and secretory tissues Implicated in disease-related processes, including prostate cancer progression	[4,25]
**FABP12**	Retina, testis	PPARγ signaling and altered fatty acid utilization Associated with cancer progression	[13]
**FABP3/5/7**	Brain	PUFA, RXR, PPAR, ECS function and transport Neurodevelopment and neurotransmission	[1,9,22]

### 1.2. Role of Fatty Acid-Binding Proteins in Neurochemistry

#### 1.2.1. Endocannabinoid Signaling

The endocannabinoid system (ECS) is a dynamic neuromodulatory network composed of endogenous lipid signaling molecules, receptors, metabolic enzymes, and transport proteins such as FABPs that regulate neuronal excitability and synaptic transmission [1]. The primary ECS receptors, named cannabinoid receptor type 1 (CB1) and type 2 (CB2), are Gi/o-coupled G-protein-coupled receptors that modulate intracellular signaling pathways following activation by dampening signaling [26]. CB1 is mainly expressed in the CNS, whereas CB2 is expressed in the peripheral immune tissue [27]. However, CB2 has also recently been found in lower amounts in the brain [28]. In neural tissues, CB1 and 2 activation modulates both calcium and potassium channels, resulting in decreased firing [29,30]. Being relatively widely expressed in presynaptic terminals of neurons [31], CB1 and 2 act as retrograde signaling receptors to control neuronal excitability and therefore development and influences widespread excitatory and inhibitory firing rates, making it a powerful neuromodulating system [32]. ECS can also be exogenously activated via Δ9-tetrahydrocannabinol (THC), the main psychoactive component in cannabis [1,33].

**Figure 1:**
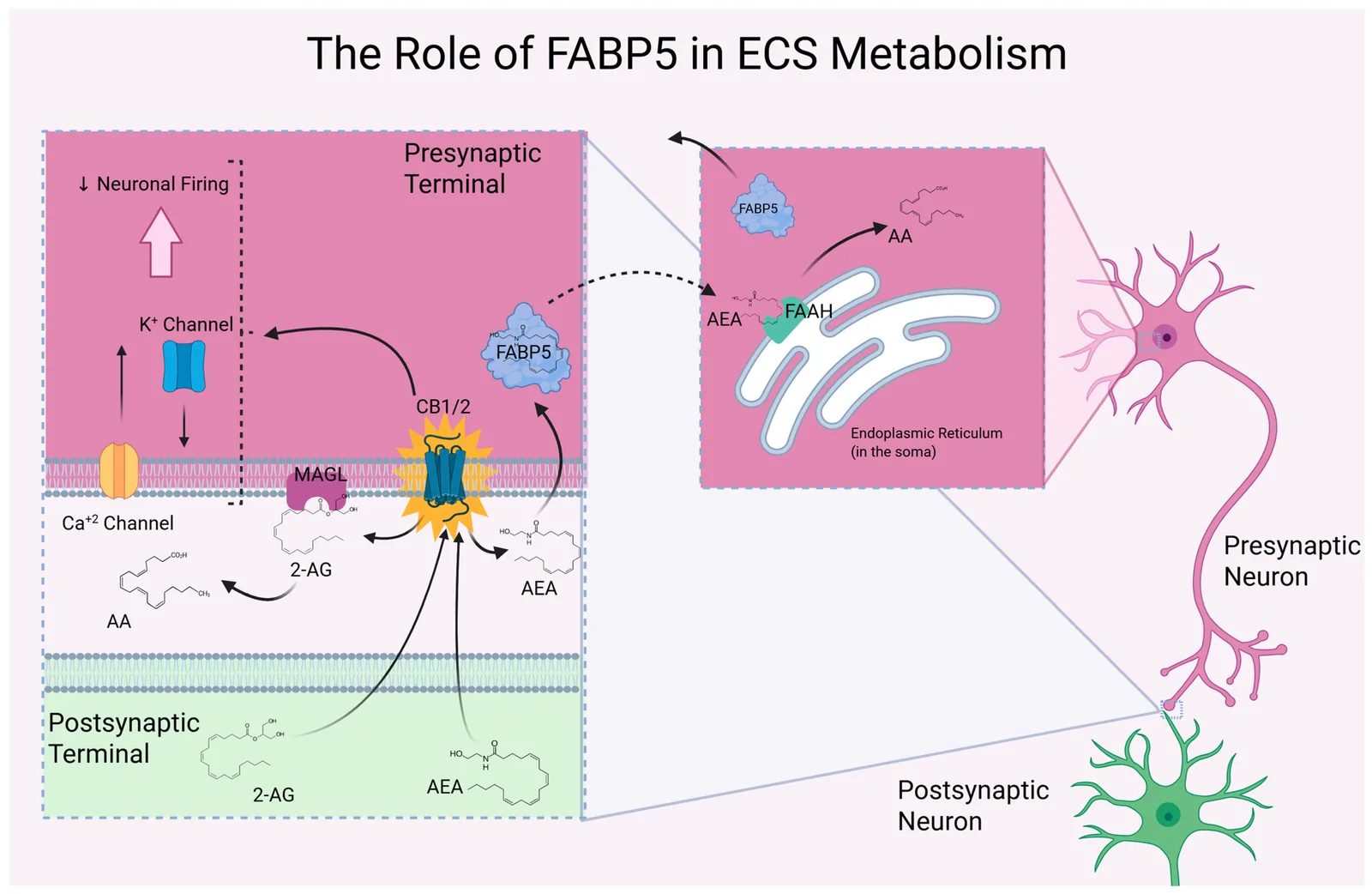
The role of FABP5 in ECS metabolism. The figure depicts the basic mechanism of the ECS in neurons. 2-AG and AEA are released by the postsynaptic neuron as retrograde signaling molecules. The two ligands bind to Gi/o-coupled CB1 or CB2 GPCRs, modulating presynaptic neuronal firing by influencing ion channel activity. AEA will diffuse into the cell where FABP5 subsequently traffics AEA to the soma, to be cleaved by FAAH expressed on the endoplasmic reticulum (ER). FAAH is shown to be expressed in the presynaptic neuron but can be cleaved in the same way by the postsynaptic neuron if FAAH and FABP5 are expressed.

Endogenously, cannabinoid receptors are activated by either anandamide (AEA) or 2-arachidonoylglycerol (2-AG). These molecules are classified as eicosanoids, meaning they are lipid-based signaling molecules derived from PUFAs [34,35]. AEA and 2-AG are cleaved and degraded by fatty acid amide hydrolase (FAAH) and monoacylglycerol lipase (MAGL), respectively. FAAH is expressed intracellularly, mainly on the endoplasmic reticulum [36], whereas MAGL is expressed on the cellular membrane on presynaptic cells, directly cleaving 2-AG to arachidonic acid (AA) [27,37]. Given the expression of FAAH inside of the cell and the lipophilic nature of AEA, intracellular transport proteins are required to facilitate AEA trafficking for enzymatic degradation. FABP5 is critical for bringing AEA to FAAH for degradation into AA and ethanolamide, and its removal results in intracellular accumulation of AEA [32,36,37]. A graphical representation of the role of FABP5 in endocannabinoid metabolism can be seen in Figure 1. While FABP7 is also localized in the brain, its function seems to modulate overall levels of endocannabinoids and exogenous cannabinoids but does not show a clear link to AEA/FAAH degradation mechanisms [1].

In the context of substance use, the neurons that are most directly involved in reward are those which release dopamine. Expression of CB1 and 2 receptors on inputs to these dopaminergic neurons are largely inhibitory and act as dampening inputs onto dopaminergic pathways. In this fashion, the inhibitory action of CB1/2 activation on the inhibitory inputs to the dopamine pathways effectively disinhibit dopaminergic release, resulting in an increase in pleasure and attention [38,39,40,41]. This effect on dopamine via CB1 and 2 suggests the ECS is heavily involved in adaptive selection of not only reward-related cues, but aversive cues [39]. Preclinical evidence thereby suggests direct ECS inhibition or indirect inhibition of ECS involvement can attenuate consumption of ethanol, nicotine, opioids, cocaine, and methamphetamine [42,43,44,45,46,47,48,49]. With this in mind, the modulation of the ECS activation via AEA and 2-AG by FABPs 5 and 7 could potentially show promising effects in clinical settings, though they should be further investigated by preclinical studies.

#### 1.2.2. Peroxisome Proliferator-Activated Receptor Signaling

There are three subtypes of peroxisome proliferator-activated receptors (PPARs), all of which are located on the nucleus and act as transcription factors: PPARγ, PPARα, and PPARβ/δ [50]. The PPAR signaling cascade is similar to other nuclear receptor mechanisms, where PPARs must dimerize with co-receptor RXRs to bind to a response element, named PPAR response elements (PPREs), in promoter regions of target genes [50]. PPARα and PPARβ/δ are ubiquitously expressed throughout the brain, with PPARβ/δ having the highest expression in corticolimbic structures such as the prefrontal cortex (PFC) and nucleus accumbens (NAc) [51]. Alternatively, PPARγ expression is sparser, largely localized to the PFC and glial cells, particularly microglia and oligodendrocytes [51,52]. Various lipid-derived molecules, including fatty acids and their derivatives, can act as ligands for PPARs. The activation of PPAR signaling in the brain can modulate neuro/glial cell metabolism, cellular activity, and inflammatory processes particularly through modulation of microglia function [1,52]. In particular, dopaminergic transmission can be influenced by PPAR action. As a result, there is a myriad of promising preclinical evidence of PPAR agonism to modulate addiction and reward-based behavior, offering promising behavioral outcomes for addiction therapies. In this fashion the agonism of different PPARs has been shown to show favorable effects in reducing consumption of food, nicotine, ethanol, cocaine, opioids, and Methamphetamine [53,54].

**Figure 2:**
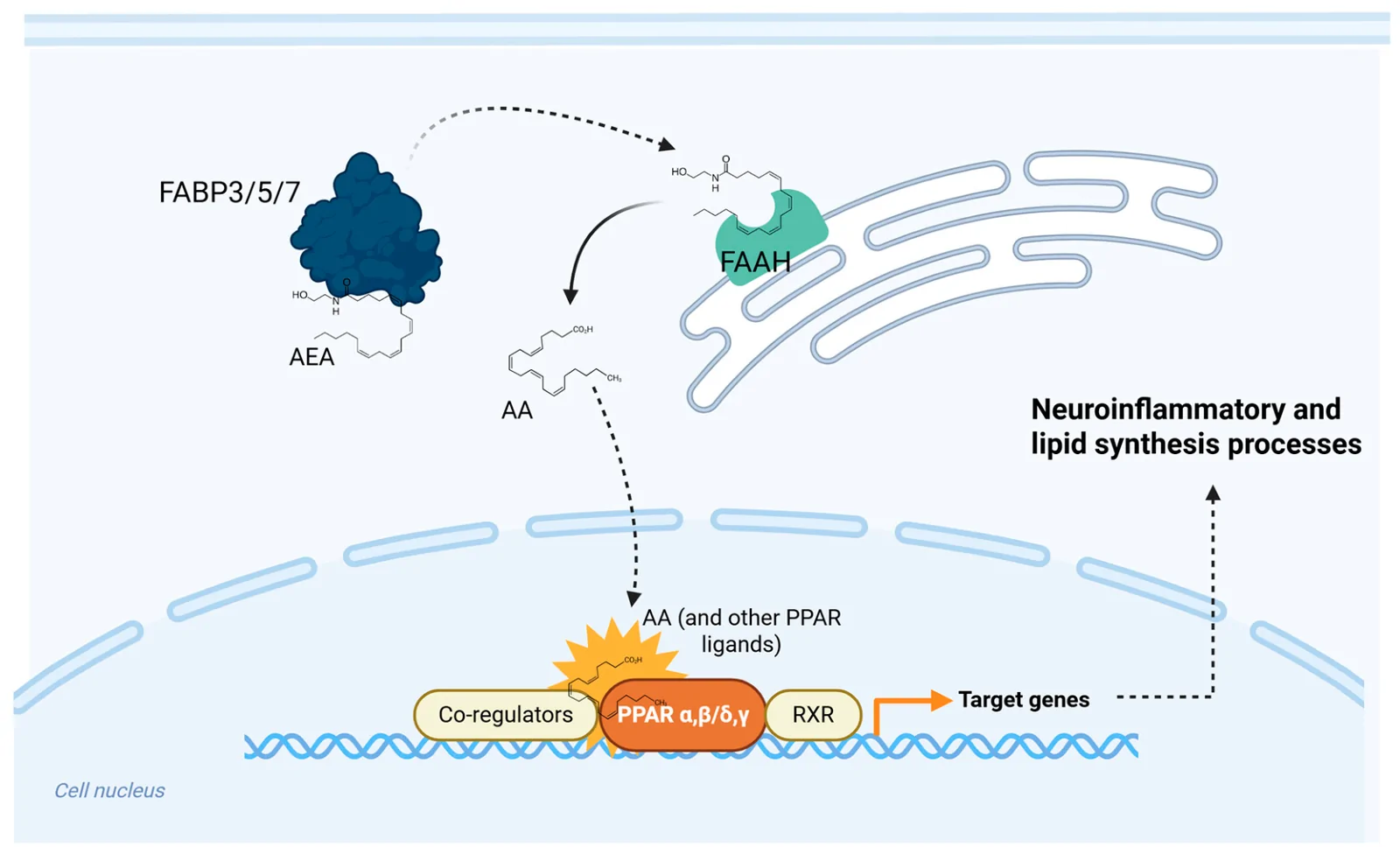
The role of FABPs in PPAR signaling. PPARs dimerize with RXR and associated co-regulatory proteins within the nucleus to regulate gene expression. PPAR ligands are all lipophilic, where the 3 CNS-expressed FABPs, FABP3, FABP5, and FABP7 can all aid in trafficking PPAR ligands within the cell to the nucleus to bind with PPAR.

It is suggested that PPARβ/δ’s main role is to act as a “nexus” for PPARγ and PPARα, influencing the expression of these two subtypes [55]. Given the ligand role of lipid molecules in the PPAR signaling system, it has been suggested that AEA can act as a ligand for PPARβ/δ [56,57]. However, more recently, it has been shown that arachidonic acid (AA), the main metabolite of AEA, may represent the primary endogenous activator. With the involvement of FABP5 in AEA metabolism [36], it is clear that there is crosstalk between the ECS and PPAR signaling system. In this fashion, FABP5’s cognitive function is shown to be mediated by PPARβ/δ activation via AA [58]. Furthermore, it can also be inferred that FABP5’s role in PPARβ/δ signaling can influence the other isoforms [55]. On the other hand, PPARγ’s main roles are in neuroinflammation and lipid regulation [51,52]. It is also known that PPARγ interact with brain-expressed FABP 3, 5 and 7 [59], suggesting that signaling systems associated with these FABPs, including the ECS, can additionally influence neuro-inflammatory and lipid synthesis processes (Figure 2). Given the role of FABPs within the PPAR system and ECS, it can be suggested that there is a crosstalk of the ECS and PPAR systems, both modulating reward processes and therefore behaviors. Although speculative, the current literature suggests that there is mechanistic promise that FABP modulation could offer, as evidenced through subsequent PPAR/ECS effects.

#### 1.2.3. Polyunsaturated Fatty Acid Uptake

**Figure 3:**
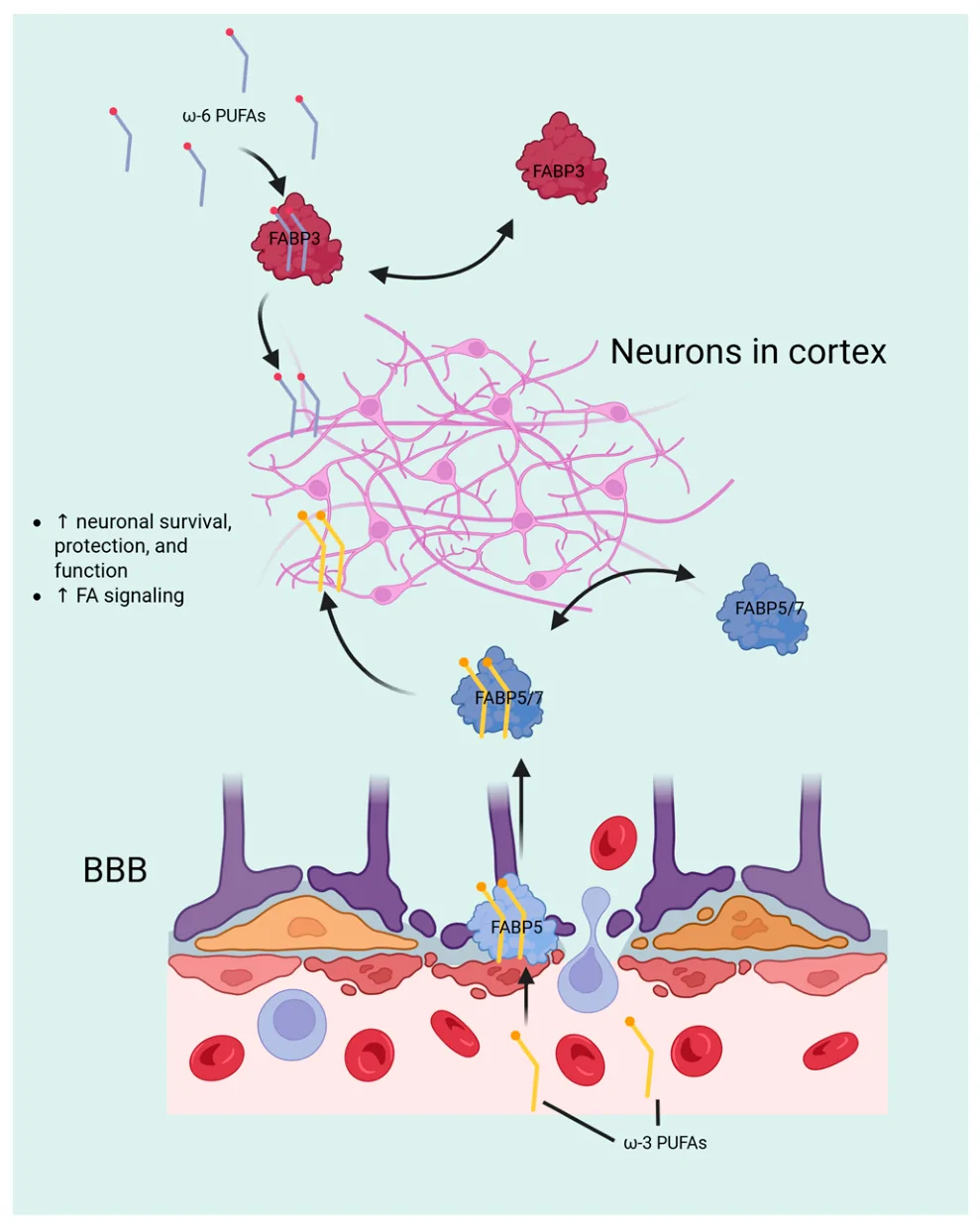
Role of FABPs in PUFA trafficking. FABP3, FABP5, and FABP7 can function as trafficking proteins for PUFAs, which are essential in basic neuronal function and survival. FABP5 aids in ω-3 PUFAs (e.g., DHA) uptake into the CNS from the BBB and further aids in carrying them to neurons along with FABP7. FABP3 has high affinity for ω-6 PUFAs (e.g., AA) and assists in their neuronal uptake.

PUFAs are long-chain fatty acids which include carbon double bonds at specific positions that are essential for neural health, development, and brain function [1,60,61]. Most notably, ω-3 and ω-6, which denote the locations of the carbon double bonds at the 3rd and 6th carbons from the end (ω carbon), are abundant in brain tissue and are the most well studied, having functions that aid in neuronal survival and neural activity. These include ω-3 PUFAs such as alpha-linolenic acid, eicosapentaenoic acid (EPA), and docosahexaenoic acid (DHA), as well as ω-6 PUFAs like linoleic acid (LA) and AA [1,60,61,62]. Deficiencies in PUFA are associated with neuro-inflammatory and neurodevelopmental disorders such as depression, anxiety, attention-deficit/hyperactivity disorder, autism spectrum disorder, dementia, Huntington’s disease, schizophrenia, and mild cognitive impairment [63,64]. In the largely hydrophilic physiological conditions within the CNS, FABP3, 5, and 7 are largely responsible for the trafficking, and therefore functional competency of ω-3 and -6 PUFAs [1]. A graphical representation of involvement of FABPs in CNS PUFA transport can be seen in Figure 3. Along with the aforementioned role of FABP5 in AEA [36], and therefore AA signaling, FABP5 is also known to facilitate DHA uptake into the CNS [65]. The deletion of FABP5 in the context was associated with deficits in working and short-term memory [65,66]. Similarly, FABP7 is also shown to bind to DHA within astrocytes and is also responsible for overall ω-3 PUFA-related processes [1,67,68,69]. Behaviorally, FABP7 deletion in mice was found to rescue alteration in brain activity through positron emission tomography following an unpredictable chronic stress model, which suggests the protein’s involvement neuroplasticity and learning in response to chronic stimuli through ECS/PUFA interplay [69]. FABP5 and dual FABP5/7 deletions have also been implicated in learning processes in this fashion, through PUFA, ECS, and PPAR-mediated pathways [70,71]. In addition, FABP3, being mainly expressed in neuronal tissue [6,72] preferentially binds to ω-6 PUFAs, such as AA [73]. This makes FABP3 also a key player in LA/AA trafficking, contributing to the role of FABP3 in conditions such as ASD and schizophrenia [1]. In essence, the role of FABP3, 5, and 7 in fatty acid trafficking and balance in this manner ultimately influences neuronal survival and excitability through ω-3 and -6, and ECS function. The role of these CNS-expressed FABPs may be a factor in which pharmacokinetic properties of lipophilic drugs are affected within the CNS, and overall behavioral outcomes through FABP involvement in dopaminergic and endocannabinoid function.

#### 1.2.4. Role of Fatty Acid-Binding Proteins in Drug Metabolism and Elimination

The principal organs which govern pharmacokinetic properties, namely drug metabolism and excretion, are the liver and kidney. In the liver, FABP1 and FABP4 are prominently expressed and contribute to lipid transport, metabolic regulation, and inflammatory signaling [74,75]. Deficits in these FABPs, along with other fatty acid transporters, can lead to the accumulation of lipid droplets in hepatic tissue. This is a hallmark of hepatic steatosis, which is involved in the pathophysiology of non-alcoholic fatty liver disease, alcoholic fatty liver, acute fatty liver in pregnancy, and hepatitis C [76]. Additionally, FABP1 is also shown to be involved in bile formation, cholesterol homeostasis, and fatty acid metabolism. Genetic knockout of FABP1, especially in tandem with a high-fat diet, increased hepatic accumulation and biliary saturation of cholesterol, altered biliary synthesis, and decreased β-oxidation rate and fatty acid synthesis [77,78,79,80].

On the other hand, FABP4 is involved in hepatic fibrosis [75]. That is because FABP4 can aid in the trafficking of transforming growth factor β (TGFβ), which is a cytokine that can mediate inflammatory factors and influence cell survival by activating Jun-N-terminal kinases (JNK) and subsequently MAPK to influence apoptotic and immune factors and ultimately promote collagen accumulation [75,81,82]. Pharmacologic inhibition of FABP4 can ameliorate liver fibrosis via reduction in JNK and TGFβ1 signaling [83]. Similarly, knockout of FABP4 is shown to reduce hepatic fibrosis whereas overexpression potentiates it, suggesting a direct role of the gene in fibrosis [75]. Given these roles of FABP1 and 4 in the metabolic and immune physiology of hepatic tissue, these proteins may also affect the metabolism distribution, and pharmacokinetics of lipid-soluble drugs and substances of abuse, although further research is required to elucidate these effects and determine therapeutic potential.

Outside of morphological alterations in which FABPs are involved, biochemically, the metabolism of other lipophilic drugs of abuse such as opioids, cocaine, and amphetamines may be affected by alterations in FABP1 and 4 expression. For example, FABP4 expression is upregulated following ethanol metabolism through cytochrome P450 2E1 (CYP2E1)-dependent pathways, promoting lipid accumulation providing a direct mechanistic link to the aforementioned FABP4-mediated fibrosis and alcohol-induced fibrosis [84]. Alternatively, it is suggested that FABP1 facilitates intracellular trafficking of lipophilic drugs within hepatocytes, potentially influencing their metabolism and clearance. These drugs include cannabinoids, NSAIDs, fibrates, benzodiazepines, glitazones, β-blockers, and steroids [79,85,86,87,88]. Specifically, hepatic FABP1 has been shown to influence the metabolism of THC through interactions with cytochrome P450 enzymes, including CYP2C9, CYP2C19, and CYP3A, highlighting a role for FABPs in modulating drug biotransformation [89]. Generally, cytochrome P450 enzymes such as CYP3A4 are also involved in the metabolism of substances including cocaine, further emphasizing the intersection between lipid-binding proteins, hepatic metabolism, and substance-related biochemical pathways [84,86]. Although further investigations are required, it could be inferred that metabolic differences in aberrant FABP genes, or the modulation of such, may be a potential therapeutic target in which substance use could be targeted. In the same manner, caution to chronic modulation of FABPs should be raised due to their apparent roles in metabolism and overall function of metabolic organs.

FABP1 is also found in the kidney, while FABP1, 2 and 3 are also found in urine in pathological states [74,90,91]. Like FABP5 and 7, these FABPs also play a role in fatty acid signaling and trafficking and can also modify these molecules to serve their purpose [74]. Elevated urinary levels of FABP1, 2, and 3 have been proposed as biomarkers of nephropathies and renal injury, as damage to glomerular and tubular structures can result in leakage of intracellular and circulating into the urine [91,92,93]. FABP4 is particularly important, expressed in renal tissue where it is involved in injury-response and repair mechanisms via trafficking of inflammatory mediators, similar to some of its functions in the liver [92,94,95]. In this fashion, excessive accumulation or dysregulated expression of FABP4 in the cytoplasm of renal cells can exacerbate kidney fibrosis, ultimately leading to reduced kidney function and has been linked to a myriad of kidney diseases. Nevertheless, the resulting altered pharmacokinetics of drugs of abuse due to decreased excretion via renal fibrosis or reduced metabolism via hepatic fibrosis and stenosis may be a factor in which FABPs can peripherally alter drug pharmacokinetics and therefore consumption behavior. Further studies are required to elucidate these effects and again, while there is therapeutic potential, caution should be raised in the chronic modulation of FABPs, as they have roles in nearly all tissues and affect lipid metabolism and trafficking throughout the body. A summary of the role of FABPs in liver- and kidney-mediated drug metabolism and excretion can be found in Table 2.

## 2. Methods of Fatty Acid-Binding Protein Manipulation

### 2.1. Transgenic Knockout

Transgenic knockout models involve the targeted deletion of a gene of interest, allowing researchers to assess the functional consequences of a complete loss of that specific gene. Most FABP knockout (−/−) studies utilize mouse models on distinct C57BL/6 genetic backgrounds, which helped reveal the effects of chronic FABP deficiency [1,96,97]. These models help infer how missense polymorphisms may affect development and drive physiological adaptations. Effects of transgenic FABP knockout are listed in Table 3.

For example, FABP1−/− mice have been found to increase brain AEA and 2-AG levels, which supports the role of FABP1 in the modulation of endocannabinoid signaling by limiting arachidonic acid levels [98]. Another study examining FABP2−/− mice and FABP1−/− mice, after giving them high-fat diets (HFD), showed that FABP1−/− increased body fat and weight, whereas FABP2−/− decreased body weight relative to WT [99]. Interestingly, a separate study analyzing a double knockout of FABP1 and 2 in HFD mice found these opposing results to balance one another out, resulting in intermediate body weight and composition profiles [7]. The authors suggest this may be driven by opposing alterations in endocannabinoid-associated feeding behavior, where FABP1−/− showed higher endocannabinoid levels and FABP2−/− showed lower. It is also suggested that differences may be influenced by alterations in metabolic fuel utilization. These findings again highlight the potential importance of FABP-related endocannabinoid signaling in motivational and reinforcement seeking behaviors. Additionally, when examining HFD effects on FABP4−/− mice, it has been seen to reduce thermogenesis and whole-body energy [100]. FABP4−/− mice have also been seen to reduce mammary tumor growth and metastasis due to changes in inflammatory pathways, including altered nuclear factor-κB (NF-kB) activity and interleukin-6 (IL6) signaling [101]. A parallel mechanism exists within central nervous system microglia where inhibiting FABP4-dependent immunometabolism in the brain suppresses microglial NF-κB activation, [102]. In addition, genetically knockout FABP3, 5 and 7 increases analgesia and anti-inflammatory effects via a lack of uptake and metabolism of N-acylethanolamines (NAEs), which leads to the elevation of whole-brain AEA tone. The accumulation is due to decreased delivery of AEA to FAAH, resulting in attenuation of nociception via increased ECS activation [70,103,104,105]. Furthermore, FABP3−/− mice were seen to be resistant to 1-methyl-1,2,3,6-tetrahydropiridine (MPTP)-induced dopaminergic neurodegeneration in the substantia nigra pars compacta (SNpc) due to the reduced arachidonic acid mediated α-synuclein oligomerization and accumulation, resulting in improved motor function in the Parkinson’s disease model [106]. This further suggests that FABP-mediated lipid signaling can influence dopaminergic neuronal vulnerability and adaptative neurophysiological responses. Moreover, FABP7−/− disrupts the stability and functional organization of cell membranes by impairing astrocyte signaling responses which may lead to alterations of neuronal activity [107]. These observations suggest that FABP7 associated lipid-mediated signaling may influence long-term neuron–glia communication and synaptic adaptations. FABP7−/− has also been seen to reduce prepulse inhibition (PPI), which reflects a sensorimotor gating function, and neurogenesis while altering startle responses, indicating a role in sensory gating and neurodevelopment relevant to schizophrenia-like phenotypes [96,108]. FABP5−/− exhibited reduced memory compared to wild-type counterparts, which environmental enrichment rescued memory deficits in only male FABP5−/− mice [70]. Distinct from these central effects, studies using nerve injury models demonstrated that FABP8−/− mice exhibit alterations in nodal and internodal structure following nerve injury, indicating a role for FABP8 in peripheral myelin organization and remyelination processes [24].

**Table 3:** Summary of FABP transgenic knockouts. HFD, high-fat diet; ND, normal diet; (−/−), KO = knockout; DKO = double knockout; PPI, prepulse inhibition. Symbols: →, indicating or leading to; ↑, increase; ↓, decrease.

FABP Protein	Observations	Functional Outcome of Model	References
**FABP1 (−/−)**	HFD: exhibited obese phenotype, ↑ brain AEA and 2-AG levels	FABP1 regulates adiposity and weigh gain in ND, ↓ AA → modulate eCB levels	[98,99]
**FABP2 (−/−)**	HFD: exhibited lean phenotype	Role in reducing diet-induced adiposity and weight gain	[99]
**FABP1 + 2 DKO**	Body weights between FABP1KO and FABP2KO	Supports distinct functions of FABP1 and 2	[7]
**FABP4 (−/−)**	HFD: ↓ thermogenesis, ND: ↓ metastasis/tumor growth	Metabolic/thermogenic regulation, tumor metastasis/growth	[100,101]
**FABP3/5/7 (−/−)**	↑ analgesia/anti-inflammatory effects	eCB modulation → ↓ nociception	[70,103,104,105]
**FABP7 (−/−)**	↓ PPI and neurogenesis	Sensory gating/neurodev. → schizophrenia-like phenotypes	[96,108]
**FABP8 (−/−)**	Post-nerve injury alteration in nodal structure	Myelin organization and repair	[24]

### 2.2. Pharmacological Inhibition

Pharmacological inhibition represents another approach used to study FABP function and develop FABP-based pharmacological therapies. For example, FABP5 inhibitor ART 26.12 has been seen to promote periaqueductal gray and broad plasma proteomic translation [109]. In the same light, the small-molecule inhibitor SBFI26 has been seen to block pro-tumorigenic signaling pathways, which suppresses proliferation, invasion, and metastasis across multiple cancer models [110,111,112]. Inhibition of FABP5 and FABP7 using SBFI26 blocks the transport of the endocannabinoid anandamide (AEA) to fatty acid amide hydrolase (FAAH), thereby increasing AEA availability in the brain [113]. This elevation in endocannabinoid levels triggers cannabinoid receptors that produce significant pain reduction effects, which highlights SBFI26 as a potent anti-nociceptive agent with mild anti-inflammatory activity in mice [104,114]. This is consistent with the role of FABPs as intracellular lipid transporters, as overall this inhibition alters endocannabinoid signaling by limiting ligand access to metabolic enzymes and receptors. In addition to these neural FABPs, pharmacological targeting of FABP4 has been shown to influence metabolic and inflammatory pathways, highlighting a broader role for FABP inhibition in regulating systemic lipid signaling [115]. Notably, these manipulations also produce functional outcomes, as inhibition of FABPs has been associated with reduced ethanol consumption in mice, suggesting that FABPs regulate signaling processes in a manner that extends beyond simple ligand availability [115,116].

Additional inhibitors include BMS309403, which significantly downregulates FABP4 protein expression and is seen to promote locomotor recovery following spinal cord injury [117]. BMS309403 is also considered a competitive inhibitor for FABP5 and has been shown to overcome the retinoic acid (RA) resistance in UW228-2 cells, suppressing the growth of medulloblastoma cells [118]. MF6, a derivative of BMS309403, acts as a FABP5 and 7 inhibitor and has been indicated to have a powerful protective function for the mitochondria in the oligodendrocytes of experimental autoimmune encephalomyelitis (EAE) mice via FABP inhibition [119,120]. Both MF6 and FABP3/5-specific inhibitor HY11-08 (HY08) have also been found to suppress promotor activity in human neuroblastoma cells, suggesting potential neuroprotective applications [121,122]. In multiple system atrophy (MSA), which is characterized by the accumulation of misfolded α-synuclein (αSyn) in glial cells, inhibition of FABP7 using MF6 was seen to reduce α-synuclein aggregation and propagation, improving cellular and neuronal health, and ameliorated motor deficits in MSA models [123].

This was similarly seen using the FABP3-specific inhibitor MF1 (also derivative of BMS309403), where the reduction in αSyn accumulation in dopaminergic neurons lead to inhibition of oligomerization of αSyn, loss of dopaminergic neurons, and Parkinson’s disease (PD)-like motor deficits in MPTP-treated mice, making it a therapeutic candidate for synucleinopathies [124]. This inhibitor has even been seen to prevent spread of αSyn following injection of a αSyn pre-formed fibrils (PFF) into the bilateral SNpc, which seemed to ameliorate cognitive impairments compared to controls with no inhibitor present [125]. MF1 was also seen to enhance GABA currents and act as a mild positive modulator of the GABA_A_ receptor through benzodiazepine recognition sites, suggesting its anti-epileptic effects [126]. Table 4 summarizes the relevant literature on FABP inhibitors, while Table 5 lists the affinity of each inhibitor to FABP subtypes.

### 2.3. Adeno-Associated Virus Vector

Adeno-associated virus (AAV) is a versatile viral vector technology that can be engineered to deliver DNA to targeted cells [132]. It is ultimately a protein shell, surrounding a single-stranded DNA that can be modified to carry a gene of interest. When injected into a specific brain region, the virus infects local cells and induces expression of the inserted gene, allowing for targeted manipulation of protein levels. Unlike knockout models, AAV-mediated approaches enable localized overexpression or knockdown of FABP subtypes in adult animals. For example, in one study, AAV-driven overexpression of FABP5 in the basolateral amygdala (BLA) was achieved through stereotaxic intracranial injection, resulting in alterations in endocannabinoid signaling [133]. Functionally, this manipulation reduced stress-induced reinstatement of cocaine-seeking behavior, highlighting a role for FABP5 in modulating reward-related circuitry and stress responses. 

In a separate study, AAV vectors were used to restore FABP3 expression in the substantia nigra pars compacta of FABP3 knockout mice following α-synuclein pre-formed fibril injection [125]. This targeted re-expression of FABP3 led to increased phosphorylation and aggregation of α-synuclein, supporting a role for FABP3 in facilitating the spread of synuclein pathology and contributing to neurodegenerative processes [106,125]. Studies utilizing AAVs to identify brain region-specific roles of FABPs are listed in Table 6. Collectively, these findings demonstrate that AAV-based approaches enable precise region-specific investigation of FABP function, revealing distinct and context-dependent roles of FABP subtypes in both reward-related behavior and disease pathology.

## 3. Methods

A narrative literature review was conducted using searches for PubMed. Inclusion of articles required them to pertain to FABPs, the major pathways FABPs affect, methods of experimentally manipulating FABPs, FABPs’ role in reward, and FABPs’ interaction with specific psychoactive drugs. Search terms included (“fatty acid binding protein” OR “FABP”) AND (“subtypes” OR “localization” OR “biophysiology” OR “neurochemistry” OR “endocannabinoid system” OR “PPAR signaling” OR “PUFA” OR “drug metabolism” OR “manipulation” OR “transgenic knockout” OR “pharmacological inhibition” OR “AAV vector” OR “general reward behavior” OR “THC” OR “ethanol” OR “nicotine” OR “cocaine”). The final search was conducted in March 2026. The review aims to detail underlying mechanisms by which FABPs can affect addictive behavior and highlight how these proteins can serve as therapeutic targets for treating substance use disorders. Articles were included if they met the following search criteria: (1) publications written in English; (2) publication date from January 1985 to March 2026; (3) peer-reviewed articles including pediatric as well as adult population studies, rat and/or mice studies, in vitro neuronal network studies, review papers and meta-analyses; (4) outcomes related to behaviors linked to substance use or addiction directly implicating FABPs; and (5) outcomes related to non-FABP proteins were included only if FABPs were directly implicated in their mechanism of action in the full text of article and if authors deemed inclusion appropriate. Case studies and editorials were excluded. Additionally, if inclusion eligibility was unclear from the title and/or abstract, the reviewers independently assessed the full text of the articles for inclusion and exclusion criteria.

Outcomes of included studies were categorized and analyzed separately according to the type of drug-related effect assessed. Behavioral outcomes such as conditioned place preference (CPP), drug self-administration, and reinstatement of drug-seeking behavior offer information on reward, reinforcement, and relapse behaviors respectively. Autoradiography measures such as receptor binding and pharmacokinetic measures such as HPLC-MS are also measures in which drug-related effects can be examined in response to drugs of abuse.

### 3.1. Role of Fatty Acid-Binding Proteins in Substance Abuse

#### 3.1.1. Effects of FABPs on Drugs of Abuse

##### THC

FABP1, 5, and 7 have been shown to have critical roles in the pharmacokinetics of THC [66,85,134]. To reiterate, FABP1 is expressed in the liver and contributes to the metabolism of circulating THC, as evidenced by reduced rates of THC metabolism in FABP1 knockout mice [85]. Deficits of FABP 5 and 7 increased brain THC accumulation and enhanced cannabinoid-induced effects. This may be linked to the aforementioned role of FABPs in THC metabolism and indicate impaired systemic clearance. Within the CNS, both FABP5 and 7 are involved in the handling of THC and its metabolites. FABP5 promotes cellular reuptake and hydrolysis of AEA, which regulates AEA levels in the brain, reflecting that FABP5 has been proposed to deliver AEA to its metabolizing enzyme FAAH, located in endoplasmic reticulum membranes [36]. This indicates its ability to prevent excess accumulation of endocannabinoids in the brain [58]. However, FABP5 has also been recently suggested to act as a chaperone that binds to arachidonic acid (AA), a metabolite of AEA breakdown by FAAH, which facilitates its movement to the nucleus to activate and induce the expression of PPARβ/δ target genes, allowing it to potently promote learning and memory [58]. Additionally, loss of FABP5 may result in increased sequestration of THC in the brain, supporting its role in facilitating intracellular trafficking and clearance of THC [66]. In contrast, a study found FABP7 to exhibit sex-dependent effects on cannabinoid metabolism, with females displaying higher THC and metabolite levels compared to males [134]. In particular, FABP7 knockout females show reduced levels of the active metabolite 11-hydroxy-THC (11-OH-THC), suggesting that FABP7 may selectively contribute to metabolite formation or processing in a sex-specific manner [134]. A separate study revealed that genetic deletion of FABP7 decreased CB1 binding in the amygdala, secondary somatosensory cortex, and ventral caudate putamen [135]. Together, these findings demonstrate that FABPs regulate both the metabolism and distribution of THC, linking intracellular lipid-binding mechanisms to cannabinoid pharmacokinetics and downstream physiological effects (Table 7).

##### Ethanol

In the case of ethanol, the ECS is critical in the regulation of intake, craving, and overall addiction behavior, through its actions within the basolateral amygdala (BLA), NAc, and ventral tegmental area (VTA) [1,137,138]. Genetic knockout or pharmacological inhibition of CB1 [42,139], FAAH [140,141,142], and FABP5/7 [113] have all shown significant effects on the preference and consumption of alcohol. CB1 inhibition and deletion in mice can decrease ethanol preference and consumption [42,139]. In the case of FAAH, genetic deletion in mice and rats exhibits increased ethanol consumption, suggesting an inverse relationship between ECS activation/tone and alcohol reward [140,141,142]. FABP5/7 inhibition (via SBFI26), in particular, reduced alcohol preference and consumption in mice in a two-bottle choice paradigm. CB1 and FABP5/7 inhibition result in similar behavioral phenotypes, and mechanistically both result in decreased AEA binding by CB1 [1,113]. In addition, FABP5 is known to be involved in the trafficking of AEA [32,36,37] which has been suggested to have a bi-directional relationship in the modulation of reward [1]. AEA can activate CB1 receptors, but at higher concentrations is known to activate the transient receptor potential vanilloid 1 (TRPV1) receptor [143]. Consequently, the deletion of TRPV1 increased ethanol consumption in mice [144]. Taken together, the crosstalk between the inhibitory CB1 and excitatory TRPV1 via AEA mediated by FABP5 can influence the addictive action of alcohol. Potentially, pharmacological modulation of FABP5 may represent a promising therapeutic strategy for alcohol use disorders. The specific role of FABP5 and 7 in the modulation of alcohol reward pathways is evidently more complex than the previously considered role in the ECS and warrants further research [1]. A summary of the role of FABPs in alcohol consumption behavior can be seen in Table 8.

##### Nicotine

Nicotine is the main addictive constituent of tobacco products and vapes, acting on nicotinic acetylcholine receptors, and producing quick flooding of dopamine inside the brain [145]. FABP3 can be complexed with dopamine receptor 2 (D2R), where both proteins are heavily implicated in nicotine’s addictive potential [146,147,148]. Genetic deletion or pharmacological inhibition of FABP3 suppressed acquisition of chronic nicotine-induced conditioned place preference as well as relapse [146,147,149]. FABP3−/− mice completely fail to acquire nicotine CPP [147]. This behavioral change was paired with an increase in D2 positive cells as well as decreased c-fos, CaMKII, and ERK response to nicotine [147]. FABP3 inhibition with MF1 attenuated nicotine-induced upregulation of D2 in the NAc and hippocampus in mice [146]. Further, phosphorylation in CaMKII and ERK following administration of a D2 agonist was significantly reduced. MF1 also inhibited the induction of nicotine CPP [146]. Altogether these findings indicate that FABP3 may be an ideal target for modulating the DA system which could help combat addiction.

On the other hand, genetic deletion of FABP5 in mice increased nicotine-induced CPP [150], which is similar to results found for genetic deletion of FAAH [151]. This is suggested to be due to elevated AEA and 2-AG levels in the brain, which may disrupt the metabolism of nicotine and potentiate its reinforcing effects [150].

FABP4 has also been implicated in nicotine-related pathology. FABP4 upregulation in human bronchial epithelium cell culture was associated with the harmful effects of cigarette smoke, including inflammation and oxidative stress in pulmonary tissue mediated by MAPK pathways [152]. A summary of the involvement of FABPs in nicotine seeking and physiological consequences can be seen in Table 9.

##### Cocaine

Cocaine is a highly addictive substance that blocks the dopamine transporter (DAT), which directly increases dopaminergic tone. It is suggested that FABP2, 5, and 7 are associated with cocaine use and related behaviors. In the nucleus accumbens (NAc), viral vector-mediated downregulation of FABP5 in mice significantly reduced cocaine self-administration, indicating a functional role for FABP5 in reinforcing properties of cocaine [153]. Similarly, genetic deletion of FABP5 and 7 in mice reduces cocaine-seeking behavior as well as preference, even after stress-induced reinstatement, further supporting their involvement in reward and relapse-related processes [154]. Interestingly, AVV-mediated overexpression of FABP5 in the BLA also exhibited a decrease in stress-induced CPP reinstatement [133]. This implies FABP5 has region-specific effects on cocaine-seeking behavior, where global deletion and region-specific upregulation show converging effects. Peripheral markers of FABP activity have also been linked to cocaine exposure. Clinical evidence demonstrated that circulating FABP2, a marker of gut integrity, is elevated in cocaine users and is associated with increased gut permeability and microbial translocation, reflecting systemic effects of cocaine use beyond the central nervous system [155]. Overall, these findings indicate that FABPs may play a role in linking neural reward signaling with peripheral physiological changes in cocaine use (Table 10). Future studies should test if similar links can be found with other FABP subtypes or other psychoactive drugs.

- Broadly, FABP7 KO alters CB1 binding and THC metabolism, whereas reductions in cocaine seeking and ethanol consumption have been reported following combined FABP5/7 manipulation.
- FABP5 plays a divergent role depending on the drug being used. FABP5 KO blunts cocaine CPP and reduces ethanol consumption and cocaine seeking. Alternatively, BLA-selective upregulation of FABP5 also reduces cocaine seeking. However, FABP5 KO also increases brain concentration of THC and enhances nicotine CPP.
- FABP3 has been shown to alter D2R expression in response to nicotine. Involvement of FABP3 in the reinforcement of other drugs remains to be investigated; however, its interaction with D2R suggests that knockout or inhibition of the protein may be protective.
- Other FABP subtypes have been shown to play a role in drug metabolism and may be indicators of drug use; however, effects on behavioral response to substances remain to be investigated.

#### 3.1.2. Reward Deficiency Syndrome

Reward deficiency syndrome is a proposed theoretical framework suggesting that dysfunction in the dopaminergic and opioidergic reward pathways contribute to a range of neuropsychiatric conditions, including SUDs [156,157]. The RDS construct remains debated as empirical validation of its phenomenological concept is still limited [158].

FABP2 has been proposed as a marker of gut barrier permeability and linked to depressive and anxiety symptoms, as well as severity in stroke patients, suggesting a broader link between FABP-related mood and stress-related pathology relevant to RDS-associated conditions [1,159]. However, this association has been demonstrated in a non-SUD, stroke-related depression cohort, and its relevance to reward dysfunction specifically has not yet been tested. Deletions of FABP5 and 7 have been more directly linked to reward-relevant behavior, as FABP5/7−/− mice have shown increased sucrose consumption and reduced forced-swim immobility relative to wild-type controls [160]. These behavioral changes correlated with increased anandamide and related N-acylethanolamide signaling, though the precise receptor mechanism remains uncertain [160]. FABP7 deletion was correlated with increased striatal N-methyl-D-aspartate receptor binding in the striatum [71]. Striatal glutamate signaling is known to play a role in psychostimulant addiction, specifically reinstatement of CPP; thus, reduced glutamate receptor density could have implications on addiction susceptibility [161]. In the same vein, FABP5 deletions were found to alter social behaviors in a sex-dependent manner and locomotion regardless of sex, of which these effects were thought to be due to alterations in ECS signaling [71]. In other words, removing these FABPs changes how much respond to both pleasure and stress, likely by raising anandamide signaling, which highlights how FABPs may be implicated in shaping reward-related behavior. Taken together, these findings suggest FABP2, FABP5, and FABP7 may interact with reward- and stress-related circuitry relevant to the RDS framework as it applies to SUDs. However, whether FABP function actually relates to RDS-defined outcomes has not been directly tested, leaving this an open area for future research.

#### 3.1.3. Generalized Substance Use

**Figure 4:**
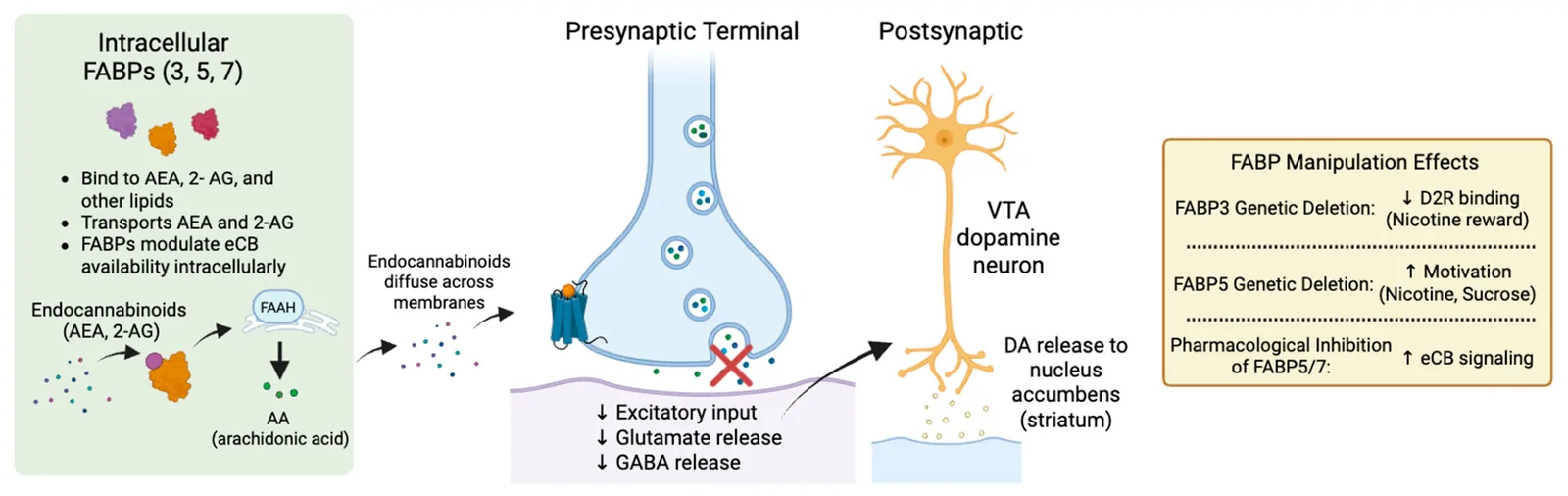
Role of FABPs in dopamine signaling. FABP3, FABP5, and FABP7 act as intracellular lipid chaperones that bind and transport endocannabinoids (AEA and 2-AG) within the cell, regulating their intracellular availability and delivery to FAAH for hydrolysis. Endocannabinoids released from the postsynaptic neuron diffuse across the synaptic membrane to activate presynaptic CB1 receptors, suppressing presynaptic neurotransmitter release (glutamate and GABA) along with synaptic input (represented by red X and downward arrows), and enhancing dopamine signaling within mesolimbic reward circuits, resulting in increased dopamine release from the ventral tegmental area (VTA) to the striatum. Genetic deletion of FABP3 reduces D2 receptor binding and nicotine reward, whereas FABP5 deletion increases motivation for nicotine and sucrose. Pharmacological inhibition of FABP5/7 enhances endocannabinoid signaling.

**Figure 5:**
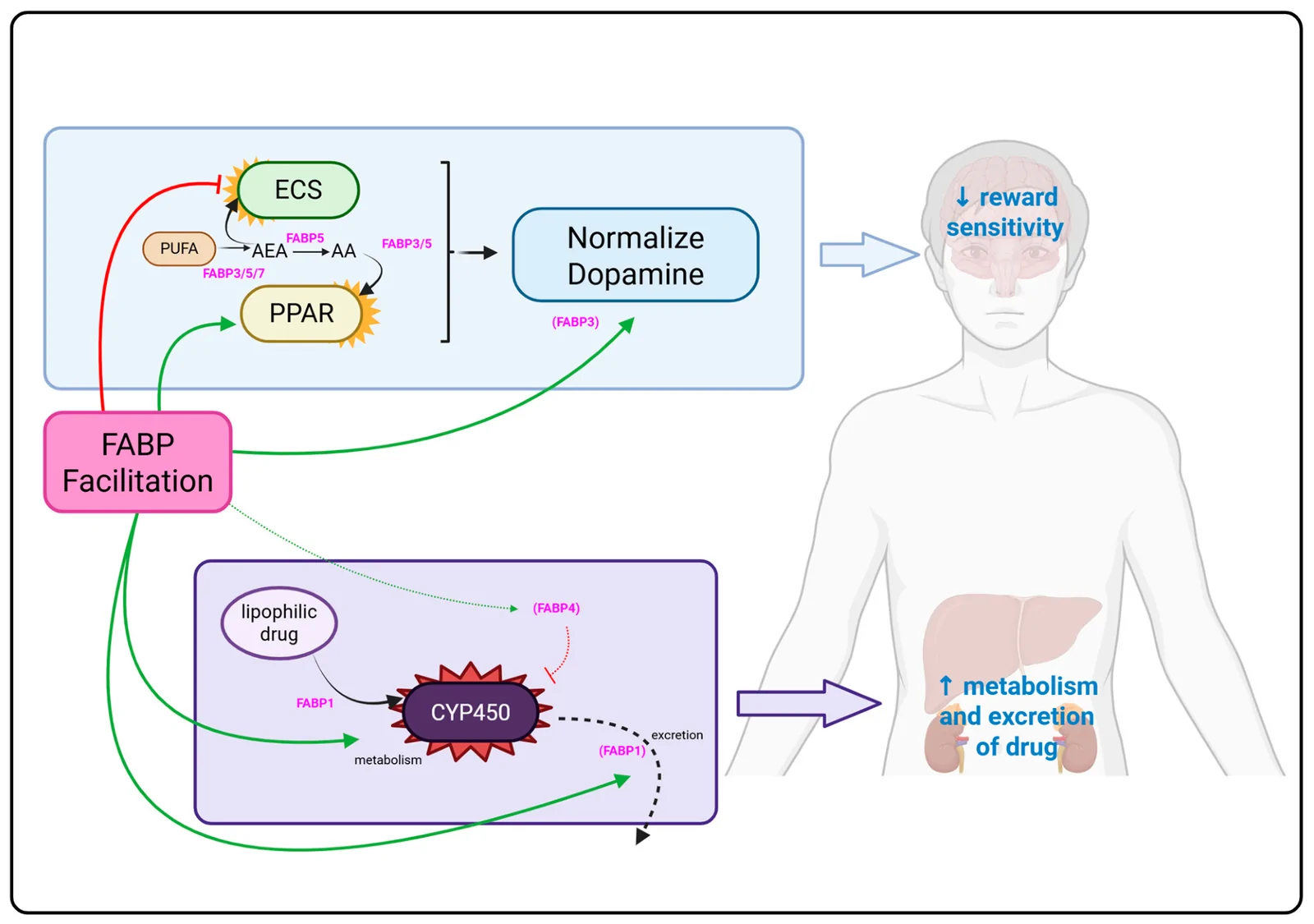
Mechanisms of FABP impact on substance use disorder. Table summarizing the major mechanisms by which FABP may affect substance use. FABPs can facilitate activation of PPARs and help normalize ECS signaling via their roles in PUFA transport. Theoretically, these mechanisms should help normalize dopamine signaling and attenuate reward sensitivity. FABPs also help modulate lipophilic transport and facilitate CYP450 enzymes in the liver and kidney which helps speed metabolism and excretion of drugs of abuse. This can help reduce toxicity of certain drugs by increasing drug elimination rate. Alternatively, rapid elimination may drive increased frequency of drug use. Since FABP4 can induce fibrosis in these tissues, chronic FABP modulation may warrant caution based on the selectivity of induction. FABPs in pink denote the mechanisms in which the specific involvement of FABPs are proposed in the literature. Parentheses denote speculative mechanisms or indirect links.

FABPs play a critical role in regulating reward-related behavior through their control of endocannabinoid signaling pathways. By facilitating the intracellular trafficking of lipid ligands such as AEA and 2-arachidonoylglycerol (2-AG), FABPs influence dopamine release within key reward regions including the ventral tegmental area (VTA) and striatum, where eCB signaling modulates glutamatergic and GABAergic inputs [1,113]. Disruption of FABP-mediated transport has been implicated as a shared mechanism underlying multiple substance use disorders, as alterations in eCB availability can shift reinforcement processes. The behavioral outcomes of FABP manipulation appear to depend on the method used: genetic deletion of FABP5 has been associated with increased motivation for nicotine and sucrose consumption, whereas pharmacological inhibition of FABP5 and FABP7 reduces alcohol intake [1,160]. Importantly, pharmacological inhibition of FABPs enhances eCB signaling without producing conditioned place preference or increasing dopaminergic signaling, suggesting a low abuse liability despite their involvement in reward-related pathways [162]. Additionally, FABP3 has been implicated in dopamine receptor-associated processes, indicating that subtype-specific differences may differentially impact reward circuitry and associated behaviors [162]. Together, these findings highlight FABPs as key modulators of reward circuitry, linking intracellular lipid transport to dopaminergic signaling and addictive behavior (Figure 4 and Figure 5).

FABPs may also contribute to compulsive drug use. While involvement in dopamine release is important for establishing the reinforcement of a substance, compulsive use describes habitual use despite adverse consequences. This is associated with severe addiction and occurs in ~20% of humans and rodents exposed to powerful reinforcers [163,164]. Compulsive drug use is best characterized by exhibition of three criteria: reduced ability to inhibit drug taking upon cue of drug availability; increased motivation for drug under progressive ratio reinforcement; and persistent drug taking despite consequences (such as food shock punishment) [165]. Several behavioral and neurochemical aberrations have been shown to predict the development of compulsive drug seeking and taking.

Compulsive use is also associated with several other changes in neurocircuitry including: increased connection between the orbitofrontal cortex and dorsomedial striatum, decreased connection between the orbitofrontal cortex and dorsomedial striatum, and impaired long-term depression in the nucleus accumbens in the context of long-term drug taking [165]. While the role of FABPs in amygdala function are well documented and have clear implications for compulsive behavior, FABPs may also play a role in these compulsion related circuits. FABP5 has been shown to alter endocannabinoid signaling in the prefrontal cortex [166], which may in turn influence noradrenergic and GABAergic transmission, though it has not been directly measured [167]. FABP5 also plays a role in modulating endocannabinoid signaling at GABAergic synapses in the striatum [168], which may have implications for LTD in the region [169].

Current treatments for SUDs are largely limited to rehabilitation services, drug replacement therapies, cognitive behavioral therapies, and pharmacological aid limited to the management of withdrawals symptoms and cravings [170]. One of a few examples of pharmacological therapies regarding SUDs is the treatment of opioid use disorder. Buprenorphine is a partial agonist of μ-opioid receptors and is used like methadone, a very-slow-acting μ-opioid receptor agonist, to provide relief to individuals experiencing opioid withdrawal [171,172]. Use of methadone and buprenorphine in the treatment of opioid use disorder replaces opioid agonism with a weaker agonist but does not completely abolish opioid signaling. In a very similar fashion, varenicline, better known as Chantix, is a partial agonist at nicotinic acetylcholine receptors and can be used to aid smoking and vaping cessation [173]. This treatment, just like buprenorphine, simply acts on the same mechanisms as the original drug in a weaker and more gradual way to curb cravings. In the treatment of alcohol use disorder, opioid receptor antagonist, naltrexone reduces the rewarding effects of alcohol. Naltrexone also reduces opioid reward though its mechanism and can induce rapid withdrawal in users who are dependent. [174,175]. Similarly, disulfiram can be used to induce sensitivity and aversion to alcohol by causing the buildup of metabolites [174]. The major issue with treatments such as naltrexone and disulfiram is patient adherence, as they induce adverse effects to curb substance use [176,177]. FABPs may be able to curb substance use without inducing rapid withdrawal as theorized mechanisms of action would not directly antagonize receptors.

Discussed therapies are generally more effective than abrupt cessation alone; most primarily target withdrawal symptoms and craving. Although speculative, we see great promise in the pharmacologic modulation of brain-acting FABPs: FABP3, FABP5, and FABP7, to counteract the decreased drive and further aid these patients in the goal of complete cessation. In this fashion, our previous findings, showing that FABP3 can interact directly with dopaminergic circuits via D2R, and showing the action of FABP5 and 7 in modulating the ECS, resulting in altered reward drive, can potentially be manipulated in a way to target SUDs from a new perspective. Individual differences in FABP gene variants may help predict susceptibility to compulsive drug use and could potentially be remedied by administration of FABP-specific pharmacotherapies. While convincing hypothetical mechanisms exist, further research is required to uncover the real-world effects of pharmacological modulation on SUDs.

#### 3.1.4. Future Research and Clinical Implications

Previous preclinical research has demonstrated that FABPs influence synaptic transmission in the context of addiction. FABPs play a role in eCB transmission and release of dopamine, GABA, glutamate, and norepinephrine in circuits involved in reward processing, stress, compulsive drug use, and reinstatement of drug seeking. FABPs have been shown to selectively modulate addictive behavior in a drug-specific manner (THC, ethanol, nicotine, cocaine). However, several gaps remain to be experimentally investigated.

The role of FABPs in the reinforcement of drugs not detailed in this review (such as opioids and benzodiazepines) remains to be investigated. Benzodiazepine reinforcement in particular is likely altered by FABPs. FABP1 attenuates diazepam metabolism by liver tissue [178]. FABP5 and FABP3 colocalize with diazepam binding inhibitor in the intestine and glia respectively [179,180]. Furthermore, FABP3 inhibition mimics the effects of benzodiazepines at GABA_A_ receptors [126]. Yet, the effect of FABP modulation on addiction behaviors such as self-administration and conditioned place preference in response to benzodiazepines or opioids have not been investigated.

While the role of FABP7 in THC metabolism has been investigated [134], the effect of other FABP subtypes and the involvement of all FABP subtypes in the metabolism of other addictive drugs is a significant gap in the literature. In this review we highlight how FABPs may alter liver- and kidney-mediated metabolism of different drugs; however, these hypotheses should be investigated experimentally. Additionally, the sex-specific effect of FABP modulation on addictive behavior has not been widely investigated despite evidence that sex influences the involvement of FABPs in THC metabolism as well as liver FABP expression and function [134,181,182].

An abundance of evidence shows that the effects of FABP in the brain are mediated, at least in part, through regulation of PUFA uptake and PPAR signaling. Despite this, most research on FABPs in the context of addiction focus on how FABPs modulate eCB signaling. Future research should interrogate the roles of each mechanism in addiction-like behavior. PPAR signaling would presumably be the most straightforward to investigate through administration of PPAR agonists (such as pioglitazone, fenofibrate, and GW501516) in combination with FABP inhibitors or within transgenic FABP knockout animals.

The previous literature relies heavily on male rodents as experimental subjects. This is a critical limitation as sex differences have been shown to alter drug consumption patterns in both rodents and humans [183,184]. The dearth of clinical studies of FABPs in addiction behavior limit the translatability of the relatively abundant preclinical research. Moreover, the design of most preclinical studies focus exclusively on reinforcement to a single substance, which ignores polysubstance use which is common in humans with substance use disorder [185]. Future studies should aim to correct these clear research gaps.

While FABP5 has been associated with human cocaine consumption [155] and FABP4 is associated with oxidative damage in smokers [152], the effect of FABP modulation on reward processing, stress, compulsive drug use, and reinstatement of drug seeking have not been tested in humans. A candidate FABP inhibitor for further research would be ART26.12, a FABP5 inhibitor which has been granted investigational new drug clearance by the FDA as a neuropathy treatment. Based on preclinical findings detailed in this review, ART26.12 may help with cocaine and alcohol abuse and may alter metabolism of THC. Further investigation on dosing, effectiveness, and abuse liability in preclinical models should preclude clinical testing as a treatment for substance use disorder. 

Beyond efficacy, the systemic distribution of FABPs raises safety considerations for long-term SUD treatment. FABP1 regulates hepatic lipid metabolism and bile acid homeostasis [77,78,79,80] while also participating in renal injury-response pathways and serving as a biomarker of tubular injury [91,92,93,94,95], suggesting that prolonged modulation of this protein warrants evaluation of hepatic and renal safety. FABP4 also functions as a circulating adipokine linked to metabolic and cardiovascular disease [18], and the lack of clinically approved FABP4 inhibitors highlights the challenges associated with translating FABP inhibition into safe therapeutic strategies [186]. In contrast, FABP5-specific inhibition has demonstrated a favorable early preclinical safety profile with limited toxicity reported to date [109,187], although these findings are largely restricted to short-term studies, and the consequences of sustained FABP5/7 inhibition on glial, neuroimmune, and metabolic function remain unknown. As per the other translational gaps discussed above, future studies should systematically evaluate the long-term hepatic, renal, metabolic, immune, and CNS consequences of chronic FABP modulation, as well as pharmacokinetic interactions and isoform-selective targeting, before FABP-directed therapies can be translated into clinical treatment strategies for substance use disorders.

## 4. Conclusions

The present review highlights the multifaceted roles of fatty acid-binding proteins in the neurobiology of substance use and addiction-related behaviors. The evidence reviewed here demonstrates that FABP modulation produces substance-specific and subtype-dependent effects on addiction-relevant behaviors in the context of THC, ethanol, nicotine, and cocaine. Despite these advances, several gaps remain. The effect of FABPs on the reinforcement of drugs such as benzodiazepines and opioids is a significant gap in the literature. Influence of FABPs on drug metabolism and sex-dependent effects, as demonstrated in FABP7 knockout studies on THC metabolism, require broader characterization across substances and subtypes. While FABPs have been shown to alter brain function through multiple pathways (PUFA, PPAR, and eCB), most addiction research has focused on the effect of FABPs on eCB signaling. Additionally, the translational applicability of current findings, predominantly derived from rodent models, has yet to be established in human populations. While a FABP inhibitor (ART26.12) is under development for pain management, its effect on addiction behavior remains to be investigated. Continued investigation into these research gaps will be essential to advance FABPs from preclinical targets to viable interventions for addiction.

## Figures and Tables

**Table 2:** The role of FABPs in drug metabolism and excretion. FABP, fatty acid-binding protein; CYP, cytochrome P450 enzyme; TGFβ, transforming growth factor β; JNK, Jun-N-terminal kinase.

Organ	FABP	Role/Effect	References
**Liver**	FABP1	Fatty acid processes; transports lipophilic drugs to CYP enzymes within hepatocytes	[77,78,79,80]
	FABP4	Implicated in inflammatory processes, aiding transport of TGFβ to JNK	[75,81,82,83]
**Kidney**	FABP1	Acts as biomarker for renal fibrosis	[79,90,91,92,93]
	FABP2		
	FABP3		
	FABP4	Implicated in inflammatory processes, aiding transport of TGFβ to JNK	[92,93,94,95]

**Table 4:** Summary of pharmacological FABP inhibitors. Symbols: → leads to; ↑ increase; ↓ decrease.

Inhibitor	Target FABP(s)	Key Observation	Functional Outcome	References
**SBFI26**	FABP5/7	Blocks AEA transport to FAAH → ↑ AEA and CB1 activation	Inhibits eCB trafficking → ↑ CB1 signaling → analgesia	[104,113,114,127,128]
**SBFI26**	FABP5	Blocks lipid signaling	Suppressing tumor progression	[110,111,112]
**BMS309403**	FABP4,5	Downregulates FABP4; improves locomotor recovery	Alters lipid signaling; reduces FABP5-mediated RA resistance	[117,118]
**MF 6**	FABP7	Reduces αSyn accumulation	Limits αSyn propagation → ↓ inflammation → neuroprotection (MSA)	[119,120,123]
**MF1**	FABP3	Enhances GABA currents	Mild positive GABA_A_ modulator (via benzodiazepine site) → anti-epileptic; ↓ αSyn	[2,124,125]

**Table 5:** FABP inhibitor binding affinities. K_d_ is a pharmacological parameter that describes the concentration of a ligand present in which 50% of available receptors are occupied. This parameter is inversely proportional to the affinity of the ligand to the receptor. Therefore, the smaller the value, the stronger the ligand binds to the receptor.

Inhibitor	FABP3 Affinity (K_d_ in µM)	FABP4 Affinity (K_d_ in µM)	FABP5 Affinity (K_d_ in µM)	FABP7 Affinity (K_d_ in µM)	Reference
**SBFI26**	-	-	0.9	0.4	[129]
**BMS309403**	-	0.552	3.02	-	[119,130]
**MF1**	0.334	0.229	0.324	3.34	[119,130,131]
**MF6**	1.04	0.530	0.874	0.02	[119,130,131]

**Table 6:** AAV techniques for FABP modulation. AAV, adeno-associated virus; BLA, basolateral amygdala; FABP, fatty acid-binding protein; SNpc, Substantia nigra pars compacta. Symbols: ↑ increase; ↓ decrease.

Protein	Manipulation (AAV)	Effect	References
**FABP3**	re-expression (SNpc, in KO mice)	↑ α-synuclein pathology and dopaminergic neurodegeneration	[125]
**FABP5**	overexpression (BLA)	↓ stress-induced cocaine CPP	[133]

**Table 7:** Summary of FABP effects on THC. THC, tetrahydrocannabinol; FAAH, fatty acid amide hydrolase; MAGL, monoacylglycerol lipase. Symbols: ↑, increase; ↓, decrease.

Protein	Manipulation	Effect	References
**FABP1**	Knockout	↓ rates of THC metabolism	[85]
**FABP5**	Knockout	↑ accumulation of THC in the brain	[66]
**FABP7**	Knockout	↓ levels of the active metabolite 11-hydroxy-THC (11-OH-THC)	[134]
**FABP7**	Genetic Deletion	↓ CB1 binding in the amygdala, secondary somatosensory cortex, and ventral caudate putamen	[135]
**FAAH and MAGL**	Inhibition (JZL195)	↓ THC withdrawal symptoms	[136]

**Table 8:** Role of FABPs in ethanol addiction. FABP, fatty acid-binding protein; CB1, cannabinoid receptor 1; FAAH, fatty acid amide hydrolase; TRPV1, transient receptor potential vanilloid 1. Symbols: ↑ increase; ↓ decrease.

FABP Subtype and Related Genes	Manipulation	Effect	Reference
**FABP5 and 7**	Inhibition (SBFI26)	↓ ethanol consumption	[113]
**CB1**	Knock out Inhibition (SR141716)	↓ ethanol consumption	[42,139]
**FAAH**	Knockout Genetic replacement with SNP rs324420 (reduced activity) Inhibition (URB597)	↑ ethanol consumption ↓ reinstatement-induced consumption	[140,141,142]
**TRPV1**	Knockout	↑ ethanol consumption	[144]

**Table 9:** Role of FABPs in nicotine addiction. FAAH, fatty acid amide hydrolase; CPP, conditioned place preference; D2R, dopamine receptor 2. Symbols: ↑ increase; ↓ decrease.

FABP Subtype and Related Genes	Manipulation	Effect	Reference
**FABP3**	Knock out	↓ CPP, **↓** D2R	[146,147]
**FABP4**	Upregulation	↑ oxidative stress in smokers	[152]
**FABP5**	Knock out	↑ nicotine CPP	[150]
**FAAH**	Knock out	↑ nicotine CPP	[151]

**Table 10:** Summary of FABP effects on cocaine. FABP, fatty acid-binding protein; KO, knockout. Symbols: ↑, increase; ↓, decrease.

Protein	Correlation with Condition	Results	References
**FABP2**	Cocaine usage	↑ FABP2 in cocaine users	[155]
**FABP5**	Cocaine self-administration	FABP5 (downreg.) = ↓ cocaine self-admin	[153]
**FABP5**	Stress-induced cocaine seeking	FABP5 (upreg. in BLA) = ↓ stress-induced cocaine CPP	[133]
**FABP5/7**	Cocaine seeking	FABP5/7 deletion = ↓ cocaine seeking/CPP	[154]

Summary of Substance-Specific Interactions with FABPs.

## Data Availability

No new data were created or analyzed in this study. Data sharing is not applicable to this article.
